# Reduced Virulence of an Extensively Drug-Resistant Outbreak Strain of *Mycobacterium tuberculosis* in a Murine Model

**DOI:** 10.1371/journal.pone.0094953

**Published:** 2014-04-14

**Authors:** Kristen L. Jurcic Smith, Divey Saini, Svetoslav Bardarov, Michelle Larsen, Richard Frothingham, Neel R. Gandhi, William R. Jacobs Jr., A. Willem Sturm, Sunhee Lee

**Affiliations:** 1 Human Vaccine Institute, Duke University School of Medicine, Durham, North Carolina, United States of America; 2 South Nassau Communities Hospital, Oceanside, New York, United States of America; 3 Albert Einstein College of Medicine, Yeshiva University, Bronx, New York, United States of America; 4 Emory University, Rollins School of Public Health, Atlanta, Georgia, United States of America; 5 Nelson R. Mandela School of Medicine, University of KwaZulu-Natal, Durban, South Africa; Johns Hopkins University School of Medicine, United States of America

## Abstract

Bacterial drug resistance is often associated with a fitness cost. Large outbreaks of multidrug-resistant (MDR) and extensively drug-resistant (XDR) TB have been described that predominately affect persons with HIV infection. We obtained four closely-related *Mycobacterium tuberculosis* strains (genotype F15/LAM4/KZN) from an outbreak in KwaZulu-Natal (KZN), South Africa, including drug-sensitive, MDR, and XDR clinical isolates. We compared the virulence of these strains in a murine model of aerosol *M. tuberculosis* infection for four phenotypes: (1) competitive *in vivo* growth in lung and spleen, (2) non-competitive *in vivo* growth in lung and spleen, (3) murine survival time, and (4) lung pathology. When mixtures of sensitive, MDR, and XDR KZN strains were aerosolized (competitive model), lung CFUs were similar at 60 days after infection, and spleen CFUs were ordered as follows: sensitive > MDR > XDR. When individual strains were aerosolized (non-competitive model), modest differences in lung and spleen CFUs were observed with the same ordering. C57BL/6, C3H/FeJ, and SCID mice all survived longer after infection with MDR as compared to sensitive strains. SCID mice infected with an XDR strain survived longer than those infected with MDR or sensitive strains. Lung pathology was reduced after XDR TB infection compared to sensitive or MDR TB infection. In summary, increasing degrees of drug resistance were associated with decreasing murine virulence in this collection of KZN strains as measured by all four virulence phenotypes. The predominance of HIV-infected patients in MDR and XDR TB outbreaks may be explained by decreased virulence of these strains in humans.

## Introduction

Multidrug-resistant (MDR) and extensively drug-resistant tuberculosis (XDR TB) represent a serious threat to global health [1]–[3]. MDR TB is defined by resistance to both isoniazid and rifampin. XDR TB is defined by resistance to isoniazid, rifampin, any quinolone, and at least one of three injectable drugs (amikacin, kanamycin, or capreomycin) [1]. There were an estimated 500,000 new cases of MDR TB globally in 2011, and XDR-TB has been reported by 84 countries worldwide [4]. TB cases with increasing degrees of drug resistance are associated with higher mortality, such that less than half of all XDR TB cases are curable, with one-year mortality rates as high as 85% [5]–[7]. Recently, cases with resistance exceeding the definition of XDR TB have received considerable attention, as treatment options are minimal, if even possible [8]–[10].

Drug resistance in *M. tuberculosis* is known to take place as a result of spontaneous chromosomal mutations, which confer resistance to an individual drug [11]. These resistance-conferring mutations proliferate in the setting of selective pressure from incomplete or improper treatment with TB medications. In addition to conferring drug resistance, chromosomal mutations exert a fitness cost. The magnitude and quality of the fitness cost appears to be dependent on the specific mutation and strain type. In some cases, these fitness costs curtail the pathogenicity of drug-resistant TB strains and potentially mitigate the spread of antibiotic resistance [12]–[14]. However, numerous outbreaks of MDR and XDR TB over the past two decades clearly demonstrate that MDR and XDR TB strains are readily transmissible, leading to large numbers of cases [15]–[19]. Many of these outbreaks have predominately involved persons infected with HIV and experiencing advanced immunosuppression. This could be due to the shorter latency between infection and disease seen in persons with HIV infection, reduced virulence of the drug-resistant outbreak strain, or other factors.

The evolutionary fitness of an individual strain of *M. tuberculosis* in nature is a complex characteristic determined by the bacterium's ability to infect a susceptible human host, persist and proliferate, and transmit to a secondary human host. Human tuberculosis transmission is also subject to many factors beyond the virulence of a specific strain. These confounding factors include variable host susceptibilities, variable latency from infection and disease, diagnostic delays, and efficacy of treatment. Not surprisingly, it has been difficult to elucidate the fitness of specific strains in humans [20].

We utilized a murine aerosol model to compare the virulence of closely-related sensitive, MDR, and XDR TB strains of the F15/LAM4/KZN family of *M. tuberculosis,* which has been associated with the largest outbreak of XDR TB reported globally in the KwaZulu-Natal province of South Africa [19], [21]–[23]. Over 90% of F15/LAM4/KZN XDR TB cases in this epidemic were co-infected with HIV, making it difficult to assess the ability of these strains to cause disease in HIV-uninfected human hosts. The fact that these drug-resistant KZN strains can thrive, despite the heavy burden of carrying multiple drug-resistance mutations could be explained by several possibilities, including minimal fitness cost to the resistance phenotype, an overall increase in virulence of the strain family, a relatively high transmission rate of the drug-resistant strains, or selection for the drug-resistant strains due to ineffective empiric treatment regimens.

To evaulate virulence of the drug sensitive and resistant KZN TB strains, we tested four phenotypes *in vivo*. First, we infected mice by aerosol containing mixtures of two or three strains (competitive model), and compared the relative bacterial CFU in lung and spleen at 60 days. Second, we infected mice with single strains (non-competitive model) and compared bacterial CFU at multiple time points. Third, we compared the survival of mice infected with individual strains. We used relatively-resistant C57BL6 mice, susceptible C3H/FeJ mice, and highly-susceptible SCID mice. Lastly, we compared lung pathology after infection with various strains.

## Results

### Infection-induced innate immune responses with KZN strains

**Figure 1:**
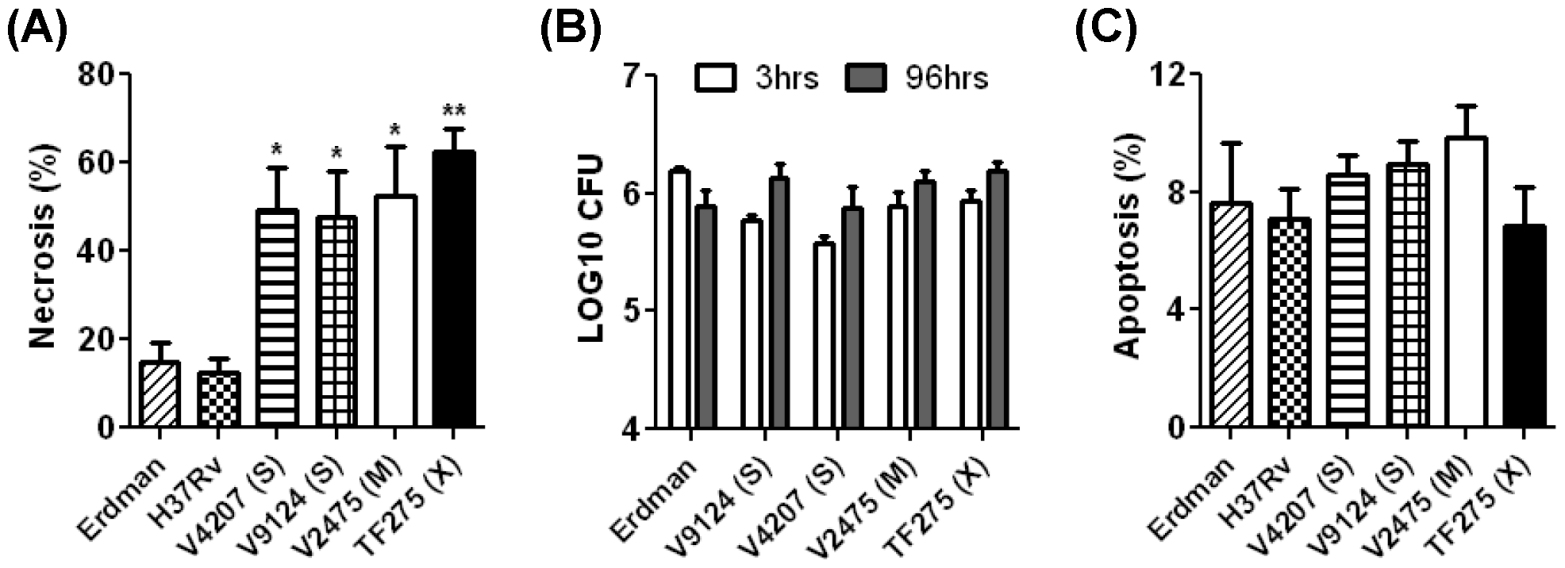
Necrosis and apoptosis induction by KZN *M. tuberculosis* strains *in vitro*. Erdman and H37Rv reference strains were compared to sensitive (S), multidrug-resistant (M), and extensively drug-resistant (X) KZN strains. (A) For necrosis measurement, alveolar epithelial cells (A549) were infected with the KZN strains at MOI of 10 for 96 h and the supernatant was assayed for lactate dehydrogenase (LDH). Percentage cytotoxicity was calculated by the following formula: [release of LDH from infected cells (OD490)-release of LDH from uninfected control/maximum LDH release (OD490)] X 100. Data from four independent experiments. (B) Infected A549 cells were lysed and plated to monitor bacterial survival. (C) The percentage of apoptosis of THP1 macrophages infected with the KZN strains at an MOI of 10 was determined by TUNEL staining of DNA fragmentation. Values are means with error bars indicating standard error. Statistically significant differences in necrosis relative to that of Erdman are shown: *, P<0.05; **, P<0.001.

In order to evaluate whether KZN strains differ in their ability to evade innate immune responses, we assessed the induction or inhibition of apoptosis and necrosis in alveolar epithelial cells (A549) infected with KZN strains and reference *M. tuberculosis* strains. We compared two drug-sensitive KZN strains, V9124 (S) and V4025 (S), a MDR KZN strain V2475 (M), a XDR KZN strain TF275, and two reference strains, Erdman and H37Rv. Whole genome analysis of these strains regarding their respective drug resistance and fitness changes have been previously reported [23]. At 96 hours post-infection, KZN strains induced a significantly higher level of necrosis than the laboratory strains, Erdman and H37Rv (Figure 1A). Interestingly, the XDR KZN strain, TF275, showed the highest level of necrosis. However, there was no significant difference in induction of necrosis among the KZN strains, irrespective of drug susceptibility. No statistical difference in CFU was observed among the tested strains after 96 hours infection (Figure 1B). We also detected varying degrees of necrosis induced by clinical isolates that are not part of the F15/LAM4/KZN family of *M. tuberculosis* (Figure S1), which confirms our finding that an innate ability to induce a significant level of necrosis by the KZN strains is not a general phenotype of all clinical isoates. Quantitation of apoptosis at 96 hours post infection showed no difference in the levels of apoptosis among the tested strains (Figure 1C). These data suggest that the KZN strains penetrate the barrier imposed by the human alveolar epithelium by increasing necrosis of infected cells, thus augmenting the establishment and transmission of infection.

### Competitive *in vivo* growth

**Figure 2:**
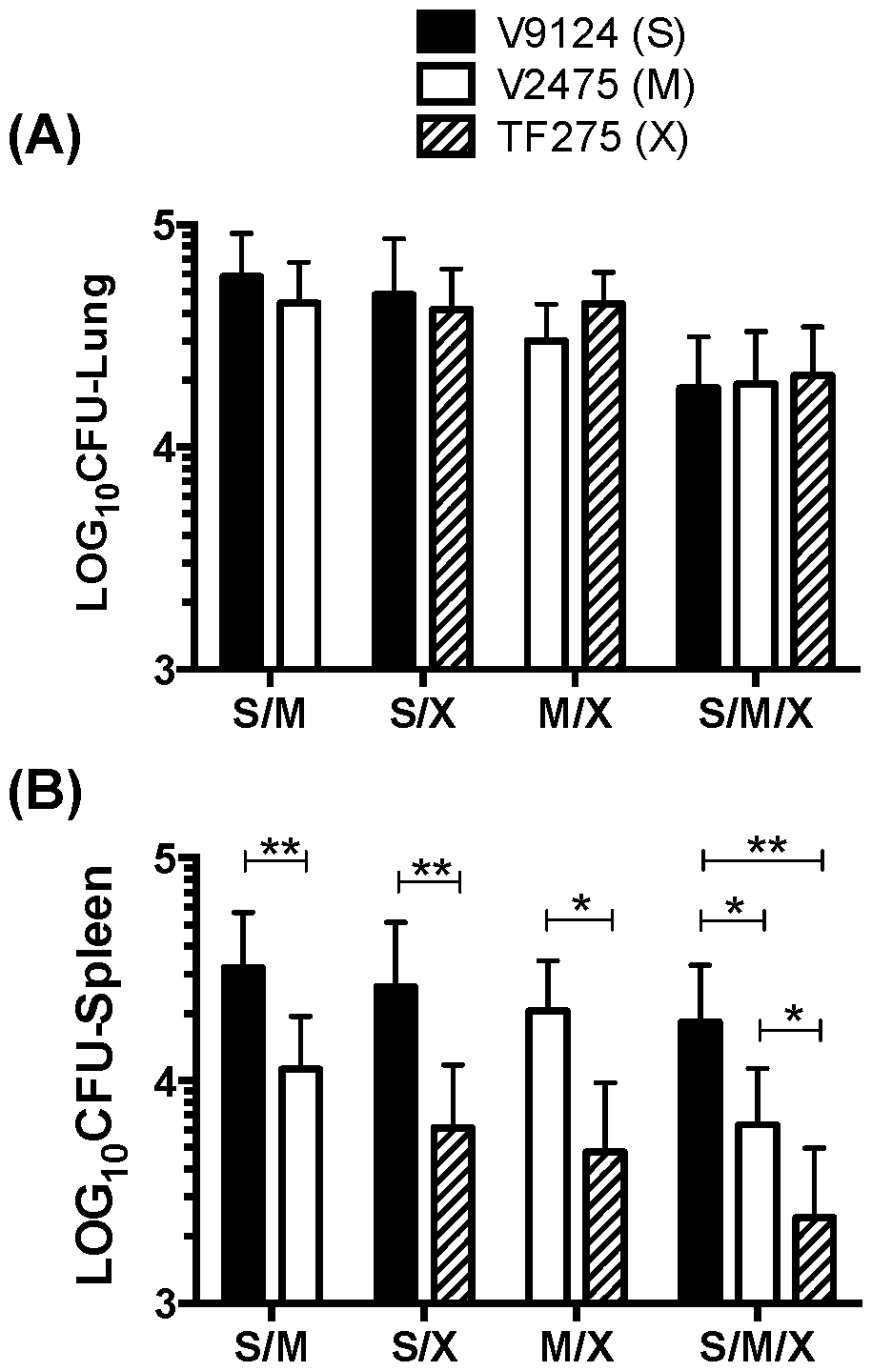
Competitive growth in murine aerosol model. Mice were aerosolized with combinations of two (n = 10) or three (n = 12) sensitive (S), multidrug-resistant (M), and extensively drug-resistant (X) KZN strains. Mice were sacrificed 60 days later, and bacterial burden determined for each strain based on CFU on selective antibiotic media. Groups were compared by the Wilcoxon signed rank test. The data shown here are from a representative experiment repeated twice. Statistically significant differences between groups are shown: *, P<0.05; **, P<0.001.

Competitive tests are frequently performed to determine the relative fitness associated with a specific drug-resistance mutation [12], [13]. We adapted an *in vitro* fitness test [24] to study the competitive capacity of V9214 (S), V2475 (M), and TF275 (X) strains *in vivo*. The number of bacilli for all groups of animals (*i.e.,* receiving mixtures of either two or three bacterial strains) was similar in the lungs at day 1 (0.27<p<1) (Figure S2). The results of two group competition studies *in vivo* demonstrated no growth disadvantage of either MDR or XDR KZN strains when they are in competition with the drug sensitive KZN strain in the lungs (0.065<p<0.67) (Figure 2A). Similarly, three-group competition did not show any advantage of the KZN drug-susceptible strain over MDR and XDR KZN strains in the lungs. However, the dissemination of both drug-resistant KZN strains to the spleens from the lungs was significantly slower than that of the sensitive strain in all competition groups (0.002<p<0.03) (Figure 2B). This result indicated that the drug-resistant KZN strains were less able to disseminate to peripheral sites, which may suggest that dissemination of bacilli to other organs is a measure of virulence of *M. tuberculosis*.

### Growth of susceptible and MDR KZN strains in the lungs and spleens of immunocompetent mice

**Figure 3:**
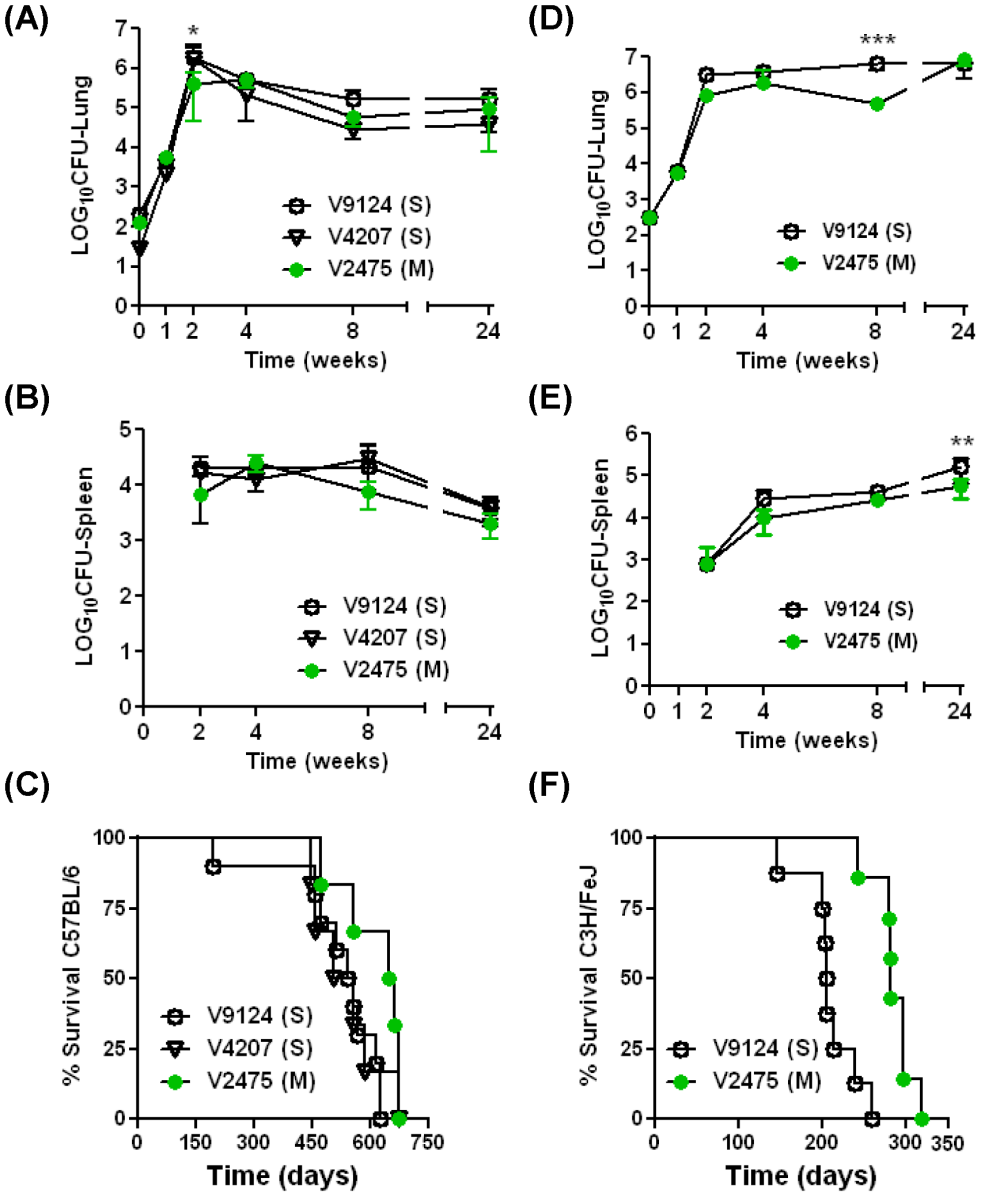
Bacterial burden and murine survival after aerosol infection. C57BL/6 mice (A, B, C) and C3H/FeJ mice (D, E, F) were infected with individual KZN strains by aerosol. The aerosolized infectious dose was determined by homogenates from 4 mice in each group at 24 hours post aerosolization. At indicated time points, four mice from each group were sacrificed and lung (A, D) and spleen (B, E) homogenates were plated on 7H10 media. CFU data are shown as means and standard errors. Spleen CFU were not assessed at week 0, and no bacteria were isolated from spleens from any mice at week 1. The survival of 8 mice from each group was compared among the infected groups (C, E). Statistically significant differences compared with V9124 (S) are shown: *, P<0.05; **, P<0.001; ***, P<0.0001.

To compare the relative virulence of the KZN strains in the murine model of tuberculosis, immunocompetent C57BL/6 and C3H/FeJ mice were infected via aerosol and monitored for bacillary growth in the tissues and for survival. The initial growth of all KZN strains, V9124 (S), V4207 (S), and V2475 (M), in *M. tuberculosis* resistant C57BL/6 mice was logarithmic, with approximately log_10_ 6.17±0.14, log_10_ 5.96±0.30, and log_10_5.41±0.27 bacilli per lung after 2 weeks of infection, respectively (Figure 3A). The difference in the number of bacilli between the two drug-susceptible strains and the MDR strain was statistically significant (p<0.05) at 2 weeks, although no statistical difference in bacterial numbers was observed among the groups in other time points. The growth of the KZN strains in the mouse spleen was also measured (Figure 3B). *M. tuberculosis* was not detected until day 14 in the spleens of C57BL/6 mice. The spleens of V9124 (S)-infected mice had a similar CFU as those of V2475 (M)-infected mice at all time points. No difference in bacterial burden was observed among the strains in either the spleens or lungs at 24 weeks (Figure 3A, 3B). Mice infected with either V9124 (S) or V4207 (S) showed accelerated mortality compared with mice infected with V2475 (M). The median survival time of mice infected with either V9124 (S) or V4207 (S) was 549 and 530 days, respectively (Figure 3C). In contrast, mice infected with V2475 (M) survived for 653 days. Survival of mice infected with an identical dose of V2475 (M) was significantly longer than those infected with V9124 (S) (p = 0.019), but not compared to mice infected with V4207 (S) (p = 0.23).

When a *M. tuberculosis* susceptible strain of mice (C3H/FeJ) was infected with the KZN clinical isolates, growth of both V9124 (S) and V2475 (M) strains were logarithmic over the first 2 weeks of the experiment, after which growth in the lungs remained steady as splenic counts increased (Figure 3D, 3E). Statistical differences of the bacterial burden in lungs of V9124 (S)- and V2475 (M)-infected mice were observed at 8 weeks post aerosolization (P<0.001). Similar to C57BL/6 mice infection, *M. tuberculosis* was not detected until day 14 in the spleens of C3H/FeJ mice, at which time almost 100-fold more bacteria were found in the spleens of C57BL/6 mice than in the spleens of C3H/FeJ mice. The bacterial counts in spleens were higher in V9124(S)-infected mice than that of V2475(M)-infected animals at week 24 (p<0.01). Similar to the difference in the bacterial burdens, survival of C3He/FeJ mice infected with V2475 (M) was significantly longer (281 days) than those infected with V9124 (S) (218 days, p = 0.0008; Figure 3F). The striking difference in the mortality of the infected mice, despite a minimum reduction *in vivo* growth rate of the drug resistant KZN strains, suggests that the V2475 (M) has yielded a degree of virulence compared to the drug-susceptible strains, possibly due to the cost associated with drug resistance.

### Growth and histopathology of an XDR KZN strain in immunocompetent mice

**Figure 4:**
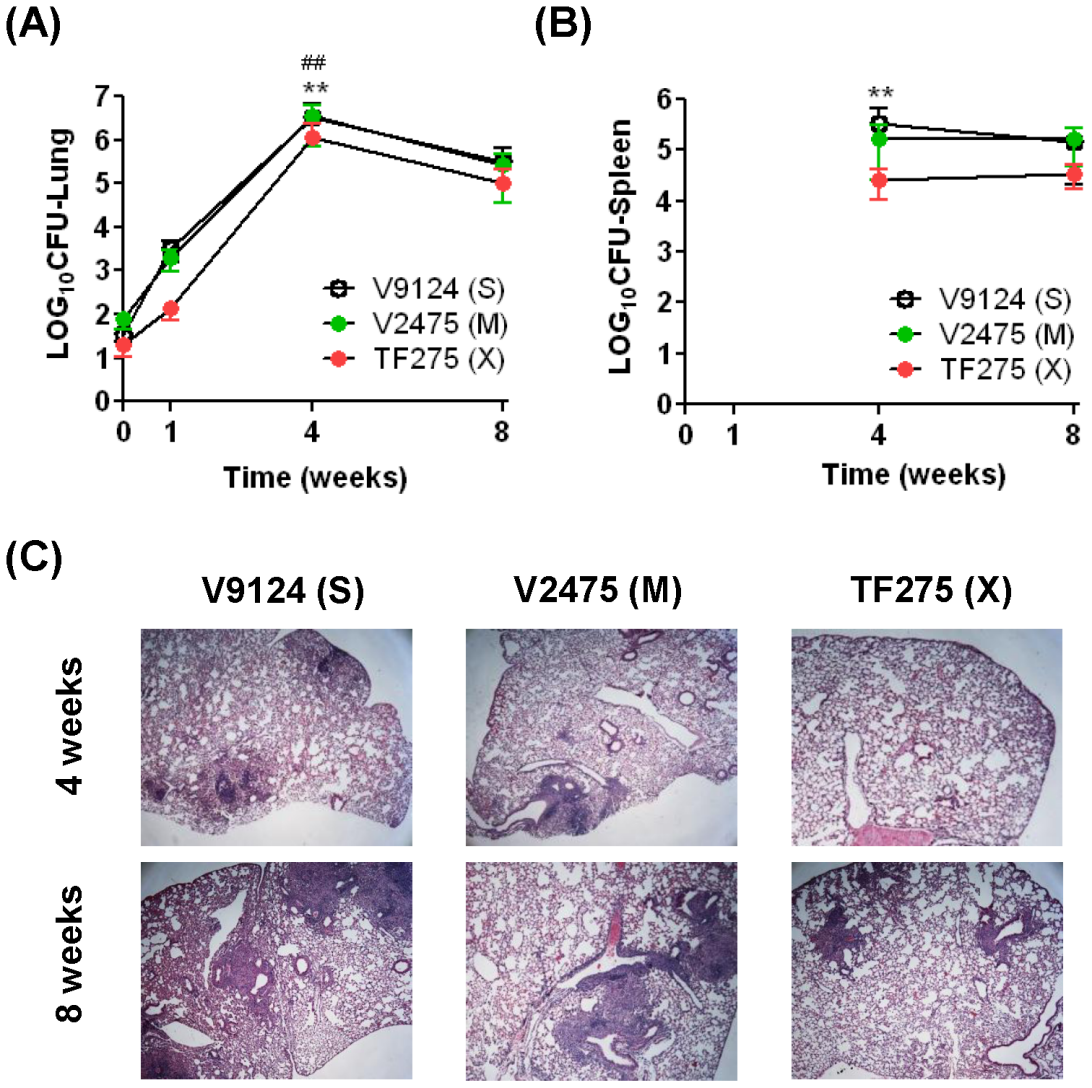
Bacterial burden and murine survival after aerosol infection. (A) C57BL/6 mice were infected with individual KZN strains by aerosol. CFU of homogenates of lungs (A) and spleens (B) were enumerated at indicated time points. Spleen CFU were not assessed at week 0, and no bacteria were isolated from spleens from any mice at week 1. Values represented are means and standard errors. (C) Cross-sections of lungs at 4 and 8 weeks post infection were stained with hematoxylin & eosin from each group. Four lungs of each group, and at each time point, were examined. One entire section of each mouse lung was evaluated. Representative lung pathology pictures are shown. Statistically significant differences between groups are shown: V9124 (S) vs TF275 (X), **, P<0.001; V2475 (M) vs TF275 (X), ##, P<0.001.

To determine whether infection with the KZN XDR strain (TF275) resulted in attenuated growth similar to the MDR strain in lungs and spleens, C57BL/6 mice were infected via the aerosol route with the TF275 (X) strain and the bacterial burden was measured (Figure 4). During the first month, rapid growth was seen for TF275 (X), with a 3.6 log_10_ increase in the lung. The bacterial burdens in the lungs of TF275 (X) infected mice were significantly lower than those of V9124 (S) and V2475 (M) infected lungs at week 4 (P<0.01, Figure 4A). After lung bacterial burdens peaked at 4 weeks, the pulmonary CFU of TF275 (X) declined to a similar number as the other KZN strains at 8 weeks post infection. *M. tuberculosis* was not detected at 1 week in the spleens. The number of bacilli in spleens of TF275 (X)-infected animals was similar to that of V2475 (M)-infected animals, but was significantly lower than that of V9124 (S) at the 4 week time point (p<0.01, Figure 4B). No difference in bacterial burden was observed at 8 weeks post aerosolization.

The possibility that the virulence of the KZN strains is related to their ability to induce a more rapid development of lung pathology, and thereby cause earlier loss of lung function, was investigated by a histological study of the lungs of mice (Figure 4C). At week 1, there was no evidence of granuloma formation in mice infected with the KZN strains, while by week 4 V9124 (S), V2475 (M), and TF275 (X) showed well-formed peribronchial granulomas. The granulomas of TF275 (X) also showed predominance of the lymphoid over the histiocytic component. By week 8, the lungs of V9124 (S)-infected mice demonstrated a slightly decreased number and size of granulomas. The granulomas of V2475 (M) infected mice were associated with epithelioid histiocytes as opposed to foamy macrophages, while the granulomas formed by TF275 (X) strain were fewer and with predominant chronic lymphoplasmacytic inflammation. Additionally, the histopathological parameters of four lungs per each group were semiquantitatively and blindly evaluated. The total score was determined by adding all subtotal numbers assigned to each parameter (Table S1). These data again demonstrated that lung pathology was reduced after XDR TB infection compared to sensitive TB infection. Analysis of *in vivo* bacterial growth after infection with TF275 (X) and histological analysis led to the conclusion that the virulence of XDR KZN strain was further attenuated compared to the MDR KZN (V2475) strain, probably due to the costs associated with additional drug resistances.

### Survival of SCID mice infected with KZN strains

**Figure 5:**
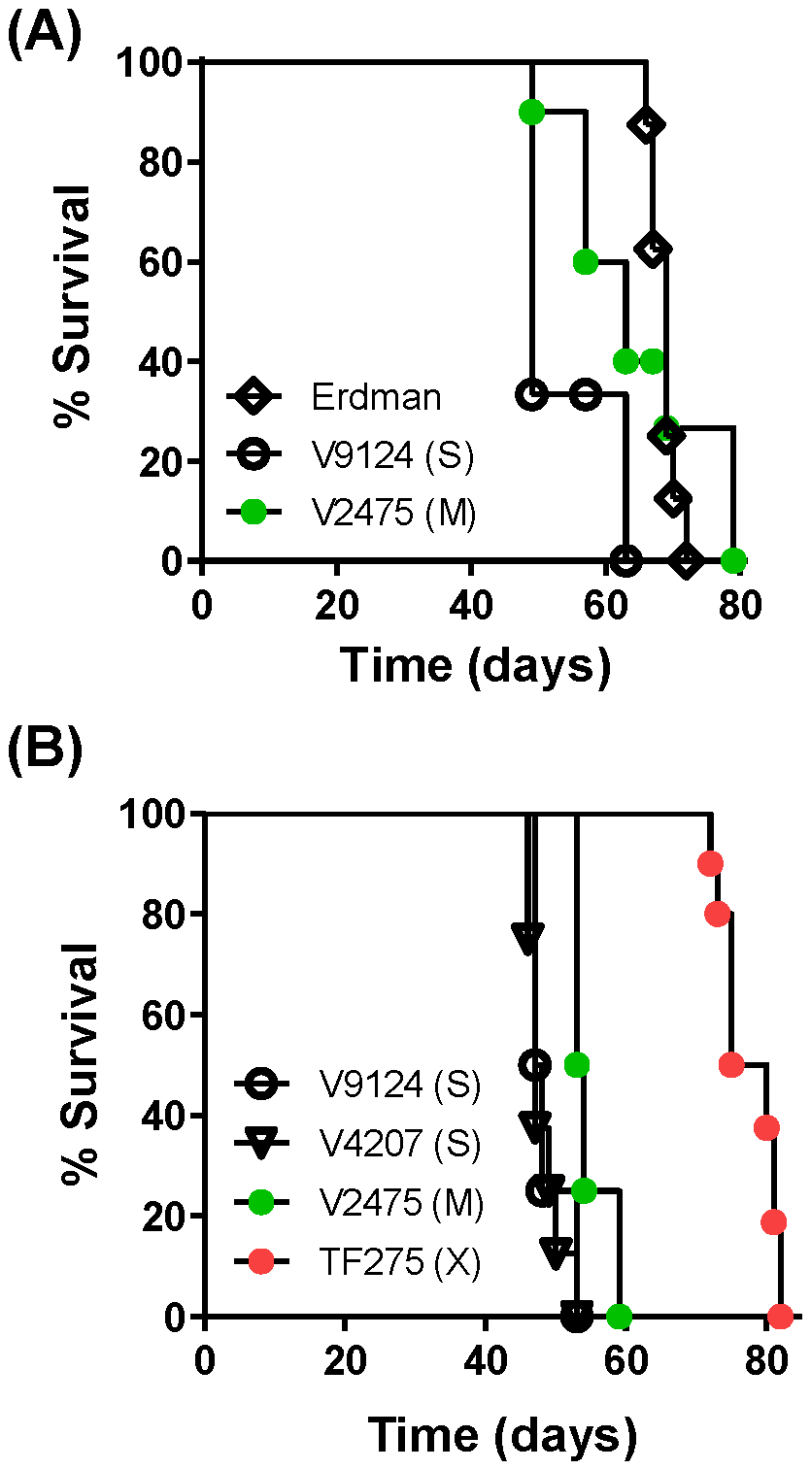
Survival of SCID mice infected with *M. tuberculosis* KZN clinical isolates. Mice were infected by aerosol with 100–200 bacilli per mouse, which was determined by lung CFU at 24 hours post infection. (A) Survival was compared among the drug-sensitive KZN strain V9124 (S), the MDR strain V2475 (M), the lab strain Erdman, and (B) the XDR strain TF275 (X). Each group has 8 mice.

Many humans infected with MDR and XDR KZN strains were also co-infected with HIV and had advanced immunosuppression. To examine the role of acquired immunity on survival of mice infected with the KZN strains, severe combined immunodeficient (SCID) mice were aerosol challenged. The first SCID experiment compared the laboratory strain Erdman to MDR (V2475) and sensitive KZN strains (V4207 and V9124). The median survival times for mice infected with Erdman (69 days) was significantly longer (p = 0.0001) than for V9124 (S) infected mice (49 days), but not significantly different from that for V2475 (M)-infected mice (63 days, Figure 5A). Mice infected with V2475 (M) survived longer than those infected with V9124 (S) (p = 0.04). All mice received similar initial bacterial doses as confirmed by CFU counts at day 1 after infection (data not shown).

The second SCID experiment (Figure 5B) evaluated the survival of the XDR KZN strain. Mice infected with TF275 (X) survived for a median of 77.5 days, which was significantly longer than the mice infected with the susceptible strains (p<0.0001) and the MDR strain (p<0.0001). SCID mice infected with two drug-susceptible strains, V4207 and V9124, succumbed with almost identical median survival times of 47.5 and 47 days, significantly shorter than for V2475 (M) infection (53.5 days, p = 0.0003 and p = 0.0012, respectively). Overall, increasing degrees of drug resistance were associated with decreased virulence in this murine model, even in immunocompromised hosts. However, the MDR and XDR KZN strains tested were sufficiently pathogenic to kill the immunocompromised SCID mice.

## Discussion

Until recently, it has been assumed that drug-resistant mutants have lower fitness and tend to spread less effectively because they will be out-competed by wild-type strains [14], [25]. However, a wider range of fitness, including the possibility that some resistant strains bear no fitness costs at all, are found using epidemiological data and *in vitro* competition studies of drug resistant bacteria [26]. Thus, the predominance of particular TB strains, such as the W/Beijing and KZN strains, among MDR and XDR TB cases in population-based and outbreak studies [27]–[29], could be due to high fitness, high transmissibility, enhanced virulence, or compensatory evolution to mitigate the fitness defects associated with drug-resistance [30].

In this study, we used *in vivo* and *in vitro* models of *M. tuberculosis* infection to examine the potential underlying mechanisms for the successful emergence of the F15/LAM4/KZN strains among MDR and XDR TB cases in KwaZulu-Natal province, South Africa. Our study investigated whether drug-resistant KZN strains have any disadvantages compared to the drug-sensitive KZN strains, as well as the Erdman and H37Rv laboratory strains. The KZN strains, irrespective of drug-susceptibility status, led to a significantly higher level of necrosis compared to laboratory strains (Figure 1a), which may facilitate invasion of pulmonary epithelial cells and contribute to successful spread among hosts. These findings build upon recent studies indicating that the spread of KZN strains may stem from stronger adhesion to, and invasion of, alveolar epithelial cells [31]. It is known that *M. tuberculosis* can actively promote necrosis over apoptosis, allowing the bacteria to evade host defense mechanisms by inducing cellular lysis and spreading infection [32].

The SCID mouse model allows evaluation of innate immune responses to TB. Infection with the drug-sensitive KZN strain V9124 induced a shorter survival time than infection with the Erdman reference strain in SCID mice. The increased necrosis associated with the KZN strains may be relevant to host defense, even without the aid of intact cellular immunity (Figure 5A), though there are certainly other differences between V9124 and Erdman. The difference in survival among the KZN strains that induced similar levels of necrosis suggests that induction of cell lysis is not the only innate mechanism determining the virulence of the KZN strains (Figure 5). No differences in apoptosis (Figure 1B), pyroptosis, or xenophagy were observed among the KZN strains (data not shown), although these cell death pathways are known to result in decreased mycobacterial survival. The involvement of other innate mechanisms and the compromised virulence mechanisms of the drug-resistant strains should be investigated to determine the basis for reduced virulence of V2475 (M) and TF275 (X) in the SCID mouse model.

In order to determine if *in vivo* growth defects were responsible for the reduced virulence and/or for the prevalence of the drug-resistant KZN strains, we analyzed the KZN strains by their competitive capacity to grow *in vivo* (Figure 2). Similar numbers of the drug-resistant and sensitive KZN strains were observed in the lungs of mice immunized with two or three strain competitions (Figure 2). This result suggests that the prevalence of the MDR and XDR KZN strains may be explained by the absence of growth defects associated with the resistance phenotype with respect to causing pulmonary disease. In addition, the similar CFU results in competitive infection of the lungs after aerosol exposure suggest that the MDR and XDR strains can be effectively transmitted. At the same time, a significantly lower number of the drug-resistant bacilli were found in spleens compared to the drug-susceptible strain in the *in vivo* competition studies. This suggests that reduced dissemination of the KZN drug-resistant strains, V2475 (M) and TF275 (X), to peripheral organs may be an important virulence trait of *M. tuberculosis* strains.

Infection of two immunocompetent mice strains, C57BL/6 and C3H/FeJ, demonstrated that KZN MDR strain (V2475) was less virulent than the KZN drug-sensitive strains, although there is no difference in growth in both the lung and spleen at 24 weeks (Figure 3). Furthermore, TF275 (X) grew similarly to V9124 (S) or V2475 (M) in the lungs and spleens at 8 weeks (Figure 4A, B). Disease severity of the XDR strain was reflected in histopathology of the lungs (Figure 4C). Thus, extended survival of infected hosts, with unchanged bacterial burdens, supports the idea that the drug-resistant KZN strains may have evolved with decreased virulence due to costs of drug resistance. Survival of immunocompromised SCID mice aerosolized with the KZN strains show that the drug-susceptible V9124 (S) strain was more virulent than the MDR KZN strain (V2475) and XDR KZN strain (TF275), similar to the survival results in two immunocompetent mice (Figure 5). These data show that the drug resistant V2475 (M) and TF275 (X) strains caused mortality in immunocompromised hosts, despite their attenuated virulence, due to their competitive growth nature.

Mathematical models predict that the MDR and XDR TB epidemics depend on transmission efficiency as well as relative fitness of drug-resistant *M. tuberculosis* compared to drug-susceptible strains. Though mice can transmit *M. tuberculosis* to other mice through coprophagia, they are not a useful model for evaluation of aerosol TB transmission among hosts. Other animal models such as guinea pigs, rabbits, or primates could be used to evaluate the relative transmissibility of the KZN strains.

In summary, we used four highly related, nearly genetically identical, *M. tuberculosis* strains that differed in drug susceptibility to perform a series of studies in a murine model of aerosol TB. There was a general coherence among the studies: The two sensitive KZN strains were more virulent than the MDR strain V2475 (M), which was in turn more virulent than the XDR strain TF275 (X). However, all strains were sufficiently virulent to induce death in the SCID mouse. The predominance of HIV-infected persons among those infected with MDR and XDR KZN strains may reflect decreased virulence of these strains in humans. Since the strains are closely related to each other, it is likely that the drug-resistance mutations cause the reduced virulence in the drug-resistant strains. However, these conclusions are limited to the four strains tested. Presently we do not know whether these results would extend to other MDR and XDR isolates from the KZN outbreak, or to MDR and XDR strains associated with other outbreaks. However, our increased knowledge and understanding of the mechanisms and effects of bacterial resistance will allow for improved epidemiological predictions, and may assist the development of new methods and strategies to combat drug-resistant TB.

## Materials and Methods

### Animals

All animal studies were approved by the Duke University Institutional Animal Care and Use Committee (IACUC) of Duke University. Specific pathogen-free C57BL/6, C3H/FeJ, and SCID mice were obtained from Jackson Laboratories (Bar Harbor, ME). After infection, we checked animal health daily for the first three days. We observed and recorded the signs of morbidity and activity (including weight, activity, gait, fur texture, posture, strength, and facial appearance) weekly until 8 months post-immunization. Animals were weighed once every 4 weeks from administration until 8 months and the frequency of weight measure was increased to weekly at the beginning of 9 months. At the initial onset of clinical signs, animals monitored and weighed daily to assess for animals that meet humane endpoints. Morbid animals that lost more than 15% of normal weight were euthanized. Morbid animals that keep normal weight received analgesia as needed, based on consultation with the veterinary medical officer. Veterinary input was obtained during the experiments in order to provide maximal relief of any distress. Any animal that experienced distress not relieved by analgesia was euthanized. Animals were euthanized by CO2 and bilateral thoracotomy or decapitation or tissue/organ collection was done as a secondary method to ensure non-recovery. Lung and spleen tissues were processed for histopathology using standard paraffin fixation, sectioning, and H&E staining. Blinded histological assessment of the stained slides was performed.

### *M. tuberculosis* strains

This study used two KZN drug-sensitive strains V9124 (S) and V4027 (S), an MDR strain V2475 (M), and an XDR strain TF275 (X). All KZN strains were recovered from patients in KwaZulu-Natal province, South Africa [23]. V2475 (M) is resistant to the first line anti-TB drugs isoniazid, rifampin, ethambutol, and pyrazinamide. TF275 (X) is resistant to isoniazid, rifampin, ethambutol, pyrazinamide, streptomycin, ethionamide, amikacin, capreomycin, kanamycin, and ofloxacin. The genomes of the strains used in this study, with the exception of V9124 (S), have been sequenced, and the detailed analysis of the sequences have been previously reported [23], [33]. The whole genome of V9124 (S) was sequenced as part of this study and the sequence was identical to that of V4207 (S), with differences in only 26 SNPs. Experiments also used *M. tuberculosis* laboratory strain H37Rv and strain Erdman. For each experiment, a vial of stock bacilli (3.6×10^8^) were aerosolized as described previously [34], [35]. The number of viable organisms in each organ sample was determined by plating serial dilutions of the homogenates.

### *In vitro* infection of THP-1 and A549 cells

THP-1 cells (TIB-202, ATCC) were treated with 8 ng/ml phorbol myristate acetate (Sigma-Aldrich) to differentiate into macrophage-like cells. THP-1 or A549 human lung adenocarcinoma epithelial cell line (CCL-185, ATCC) cells were infected with a multiplicity of infection (MOI) of 10:1 (bacteria:mammalian cells). Apoptosis of THP-1 cells (using TUNEL assay, Roche) and necrosis of A549 cells (using lactose dehydrogenase release assay, Roche) were determined at 96 hours post infection.

### Competitive fitness determined by viable cell counting

Fitness among V9124 (S), V2475 (M), and TF275 (X) strains were compared in this experiment. An identical number of bacteria from the drug sensitive strain and drug resistant strains (2.4×10^7^ of each) were mixed and aerosolized. At day 1 and day 60 the spleen and lung homogenates were plated on 7H10 media with and without appropriate antibiotics. Isoniazid (1 µg/ml) and kanamycin (5 µg/ml) were used to select for V2475 (M) and TF275 (X), respectively.

### Statistical analysis

One-way ANOVA or two-way ANOVA tests were used for analyses in which 3 or more experimental groups were compared. The log-rank test was used for the comparison of survival curves. *P* values of less than 0.05 were considered significant. Prism 5.0 software (GraphPad) was used for all analyses. For the competition study, the Wilcoxon signed-rank test was performed using SAS Software. This test does not rely on distributional assumptions of the data. The alpha level was set at 0.05 for all comparisons and no adjustments were made for multiple comparisons.

## Supporting Information

Figure S1 **Necrosis induction by various** ***M. tuberculosis*** **strains** ***in vitro*** **.** Alveolar epithelial cells (A549) were infected with the indicated clinical isolates at MOI of 10 for 96 h and the supernatant was assayed for lactate dehydrogenase (LDH). Percentage cytotoxicity was calculated by the following formula: [release of LDH from infected cells (OD490) -release of LDH from uninfected control/maximum LDH release (OD490)] X 100. Data from two independent experiments. KZN8, 9, and 10 were isolated from the KwaZulu-Natal province of South Africa and belong to Beijing lineage. InO1, InO2, and InO3 are clinical isolates of Indo-Oceanic lineage. Statistically significant differences compared with H37Rv are shown: *, P<0.05; **, P<0.01; ***, P<0.001; ****, P<0.0001.
(PDF)

Figure S2 **Bacterial load of the lungs one day post aerosolization in the three strain mixing experiement.** Bacteria in the lungs from 8 mice were measured by plating on 7H10 plates. Error bars represent standard errors. Inoculum at day one post-aerosol challenge showed no statistical differences.
(PDF)

Table S1 **Histopathology scoring of the lungs of mice 4 and 8 weeks after Infection.** The mean of the individual scores for four lungs is shown for each parameter. Histopathological parameters were semi-quantitatively and blindly evaluated. The total number of bronchi in all sections in all lungs were counted, then the number of bronchi with peri-bronchial inflammation that is either pure lymphoid or frank granulomatouse was calculated. The formula used to determine the percentage is as follows: (Number of bronchi involved X 100)/(total number of bronchi). The scoring table determined a score based on that percentage and the overall score was used to grade each strain. The same calculation was used for the vessels. Alveolitis is graded subjectively by eye by first finding the most severe form of alveolitis and then using it as a reference.
(PDF)
