# Supplementary material for: Reduced Virulence of an Extensively Drug-Resistant Outbreak Strain of Mycobacterium tuberculosis in a Murine Model
Source: PLoS One. 2014 Apr 14;9(4):e94953. doi: 10.1371/journal.pone.0094953 (PMC3986381; doi:10.1371/journal.pone.0094953)
Supplement: Figure S1 — Necrosis induction by various M. tuberculosis strains in vitro . Alveolar epithelial cells (A549) were infected with the indicated clinical isolates at MOI of 10 for 96 h and the supernatant was assayed for lactate dehydrogenase (LDH). Percentage cytotoxicity was calculated by the following formula: [release of LDH from infected cells (OD490) -release of LDH from uninfected control/maximum LDH release (OD490)] X 100. Data from two independent experiments. KZN8, 9, and 10 were isolated from the KwaZulu-Natal province of South Africa and belong to Beijing lineage. InO1, InO2, and InO3 are clinical isolates of Indo-Oceanic lineage. Statistically significant differences compared with H37Rv are shown: *, P<0.05; **, P<0.01; ***, P<0.001; ****, P<0.0001. (PDF) [file pone.0094953.s001.pdf]

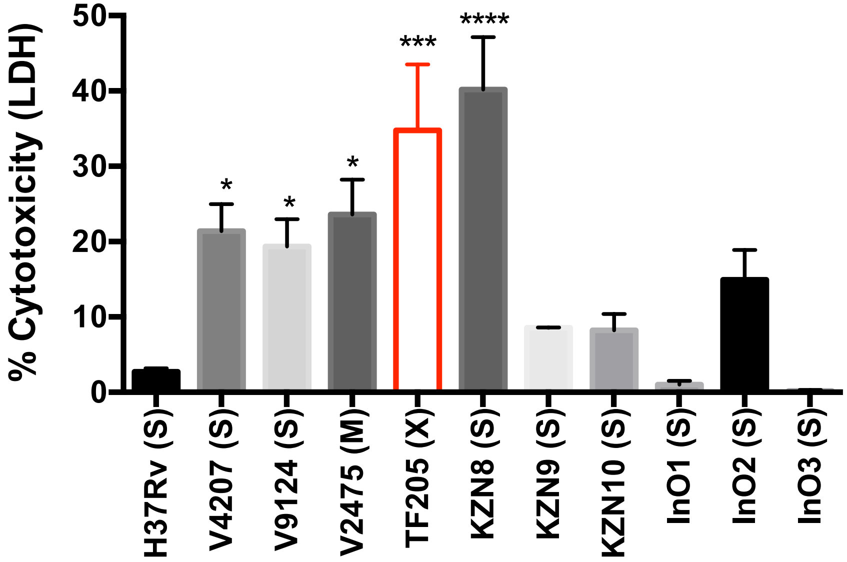

Figure S1. Necrosis induction by various *M. tuberculosis* strains *in vitro*. Alveolar epithelial cells (A549) were infected with the indicated clinical isolates at MOI of 10 for 96 h and the supernatant was assayed for lactate dehydrogenase (LDH). Percentage cytotoxicity was calculated by the following formula: [release of LDH from infected cells (OD490) -release of LDH from uninfected control/maximum LDH release (OD490)] X 100. Data from two independent experiments. KZN8, 9, and 10 were isolated from the KwaZulu-Natal province of South Africa and belong to Beijing lineage. InO1, InO2, and InO3 are clinical isolates of Indo-Oceanic lineage. Statistically significant differences compared with H37Rv are shown: *, $P < 0.05$; **, $P < 0.01$; ***, $P < 0.001$; ****, $P < 0.0001$.
