# Supplementary material for: Reduced Virulence of an Extensively Drug-Resistant Outbreak Strain of Mycobacterium tuberculosis in a Murine Model
Source: PLoS One. 2014 Apr 14;9(4):e94953. doi: 10.1371/journal.pone.0094953 (PMC3986381; doi:10.1371/journal.pone.0094953)
Supplement: Figure S2 — Bacterial load of the lungs one day post aerosolization in the three strain mixing experiement. Bacteria in the lungs from 8 mice were measured by plating on 7H10 plates. Error bars represent standard errors. Inoculum at day one post-aerosol challenge showed no statistical differences. (PDF) [file pone.0094953.s002.pdf]

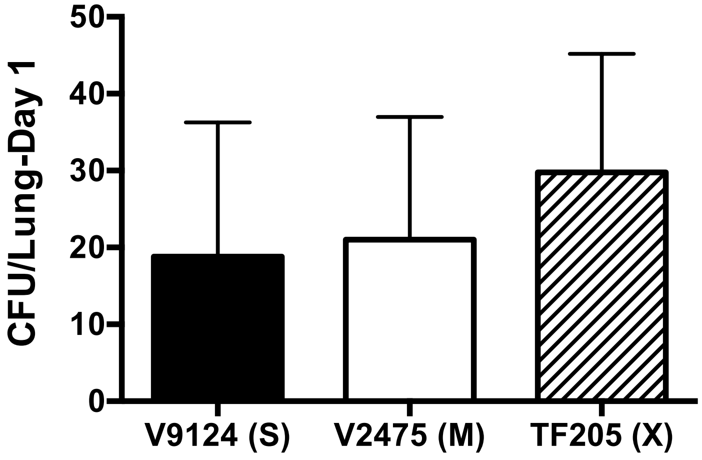

Figure S2. Bacterial load of the lungs one day post aerosolization in the three strain mixing experiement. Bacteria in the lungs from 8 mice were measured by plating on 7H10 plates. Error bars represent standard errors. Inoculum at day one post-aerosol challenge showed no statistical differences.
