# Supplementary material for: Reduced Virulence of an Extensively Drug-Resistant Outbreak Strain of Mycobacterium tuberculosis in a Murine Model
Source: PLoS One. 2014 Apr 14;9(4):e94953. doi: 10.1371/journal.pone.0094953 (PMC3986381; doi:10.1371/journal.pone.0094953)
Supplement: Table S1 — Histopathology scoring of the lungs of mice 4 and 8 weeks after Infection. The mean of the individual scores for four lungs is shown for each parameter. Histopathological parameters were semi-quantitatively and blindly evaluated. The total number of bronchi in all sections in all lungs were counted, then the number of bronchi with peri-bronchial inflammation that is either pure lymphoid or frank granulomatouse was calculated. The formula used to determine the percentage is as follows: (Number of bronchi involved X 100)/(total number of bronchi). The scoring table determined a score based on that percentage and the overall score was used to grade each strain. The same calculation was used for the vessels. Alveolitis is graded subjectively by eye by first finding the most severe form of alveolitis and then using it as a reference. (PDF) [file pone.0094953.s003.pdf]

Table S1. Histopathology scoring of the lungs of mice 4 and 8 weeks after Infection.

Strains	Peribronchiolitis	Perivasculitis	Alveolitis	Granuloma	SUM-scores
V9124-4W	1	3	3	2	**9**
V2475-4W	1	3	0	2	**6**
TF205-4W	1	2	2	1	**6**
V9124-8W	1	4	1	3	**9**
V2475-8W	1	4	1	2	**8**
TF205-8W	1	1	1	2	**5**

Scoring	
%	Score
0	0
1-20	1
20-40	2
40-60	3
60-80	4
80-100	5

* The mean of the individual scores for four lungs is shown for each parameter. Histopathological parameters were semi-quantitatively and blindly evaluated. The total number of bronchi in all sections in all lungs were counted, then the number of bronchi with peri-bronchial inflammation that is either pure lymphoid or frank granulomatouse was calculated. The formula used to determine the percentage is as follows: (Number of bronchi involved X 100) / (total number of bronchi). The scoring table determined a score based on that percentage and the overall score was used to grade each strain. The same calculation was used for the vessels. Alveolitis is graded subjectively by eye by first finding the most severe form of alveolitis and then using it as a reference.
